# Thiolation of arabinoxylan and its application in the fabrication of controlled release mucoadhesive oral films

**DOI:** 10.1186/s40199-017-0172-2

**Published:** 2017-03-20

**Authors:** Muhammad Hanif, Muhammad Zaman

**Affiliations:** Department of Pharmacy, Bahauddin Zakariya University, Multan, 60800 Pakistan

**Keywords:** Arabinoxylan, Thiolation, Thiol contents, Ex-vivo mucoadhesion, Ex-vivo permeation, Drug release

## Abstract

**Background:**

Mucoadhesion is an important property that helps oral drug delivery system to remain attached with buccal mucosa and hence to improve the delivery of the drug. The current study was designed to achieve the thiol modification of Arabinoxylan (ARX) and to develop a mucoadhesive oral film for the improved delivery of tizanidine hydrochloride (TZN HCl).

**Method:**

Synthesis of thiolated arabinoxylan (TARX) was accomplished by esterification of ARX with thioglycolic acid (TGA). TARX was further used for the development of mucoadhesive oral films which were prepared by using a solvent casting technique. Formulation of the films was designed and optimized by using central composite design (CCRD), selecting TARX (X_1_) and glycerol (X_2_) as variables. Prepared film formulations were evaluated for mechanical strength, *ex-vivo* mucoadhesion, in-vitro drug release, *ex-vivo* drug permeation, surface morphology and drug contents.

**Results:**

Thiolation of ARX was confirmed by fourier transform infra-red spectroscopy (FTIR) as a peak related to thiol group appeared at 2516 cm^−1^. The claim of successful thiolation of ARX was strengthened by the presence of 2809.003 ± 1.03 μmoles of thiol contents per gram of the polymer, which was determined by Ellman’s reagent method. From the results, it was observed that the films were of satisfactory mechanical strength and mucoadhesiveness with folding endurance greater than 300 and mucoadhesive strength 11.53 ± 0.17 N, respectively. Reasonable drug retention was observed during in-vitro dissolution (85.03% cumulative drug release) and *ex-vivo* permeation (78.90% cumulative amount of permeated drug) studies conducted for 8 h. Effects of varying concentrations of both polymer and plasticizer on prepared mucoadhesive oral films were evaluated by ANOVA and it was observed that glycerol can enhanced the dissolution as well as permeation of the drug while TARX has opposite impact on these parameters.

**Conclusion:**

In nutshell, TARX in combination with glycerolwas found to be suitable for the development of controlled release mucoadhesive oral films of TZN HCl.

**Graphical Abstract:**

Schematic diagram showing conversion of ARX to TARX, TARX to oral film and evaluation of fabricated oral film

## Background

A wide range of unmodified polymers have been used for the buccal drug delivery system as well as many other alternative routes of administration [1]. Nowadays, a newer class of the polymers namely, thiomers or thiolated polymers, are under research and development. Thiolated polymers are the polymers altered for the mucoadhesion and other additive properties due to incorporation of thiol groups in the backbone of the non-thiolated polymeric chain by replacement reactions or simple oxidation reactions [2–5]. The thiomers can help in the solubility and permeation of less water soluble and less permeable drugs [6]. They have an effective role in the development of the stronger link to mucosal membrane, which is usually developed between thiol moiety and mucin proteins. In thiomers, immobilization of thiol moiety therefore enhances the mucoadhesive strength and mucoadhesion retention time of the thiolated polymer by 2–140-folds [7, 8]. Currently thiomers have comprehensive applications as an auspicious pharmaceutical excipient in the assessing era of pharmaceutical technology [9]. Thiolated polymers along with other dosage forms, in controlled release drug delivery systems are also very effective and useful, as they provide better contact time to the drug molecules with mucosal membrane of the gastrointestinal tract as well as other routes for the drug delivery [10, 11].

Researchers involved in the formulation development are more concerned with the fabrication of optimized formulations as in order to get desired characteristics it is important to optimize the formulation. Various statistical approaches have been used in the optimization of formulation and one of them is central composite rotatable design [12]. It is a widely used statistical tool which is in use for the design and optimization of various formulations.

Different NSAIDs have been reported which give relief in pain disorder [13, 14] and are used in combination with muscle relaxant. TZN HCl is an important muscle relaxant and used in muscular spasm and pain disorders, but it has extensive hepatic metabolism and therefore its oral bioavailability is very low (about 21%). Reduced bioavailability may cause decrease in its effectiveness. Moreover, it has shorter half-life which is approximately 3 h and has dose about 2–4 mg twice daily, that can be increased to a maximum of 36 mg in 24 h. TZN HCl has p*Ka* value 8.2 and its log partition coefficient is 2.72. Characteristics mentioned above favour sustained release and mucoadhesive oral film formulation of TZN HCl [15].

Aim of the current research project is the successful thiolation of ARX and use of TARX in the development of controlled release mucoadhesive oral films. Greater retention time in the buccal mucosa will cause release of the drug in buccal cavity providing a way to absorb the drug across mucosal membrane and hence protecting TZN HCl from the first pass effect. Prevention from the first pass effect may cause the enhancement of bioavailability and effectiveness of the drug. Design and optimization of formulations was accomplished by using CCRD considering TARX (X_1_) and glycerol (X_2_) as variable factors and influence of both factors was evaluated by the application of analysis of variance (ANOVA).

## Methods

Ispaghula husk was purchased from local market of Lahore, Pakistan and it has been used for the extraction of ARX by using alkali extraction method described earlier by S. Saghir and her co-workers [16]. TZN HCl was gifted by Pharmedic Laboratories Lahore, Pakistan; Polysuccralose (sweetener) was obtained from Moringa Pharmaceuticals Pvt Ltd Lahore, Pakistan. Sodium hydroxide (NaOH), acetic acid, HPMC K15M, orange flavor and glycerol were purchased from Merck Dermstadd Germany. All the chemicals and reagents used were of analytical grade.

### Synthesis of TARX

Thiolation of arabinoxylan (ARX) was carried out by using the method already described elsewhere [17]. Briefly, TARX was prepared by developing ester linkage between ARX and thiol group provided by TGA in the presence of HCl [18]. 1 g of ARX was added in a glass beaker of 100 ml capacity, containing about 50 ml of distilled water. The final volume was adjusted to 100 ml by using the distilled water and soaked overnight at room temperature to get the polymer (ARX) completely hydrated. 2 g of TGA was added in ARX dispersion and after complete mixing of both ARX and TGA, a catalytic amount of HCl (4–5 drops) was added in it..the reaction mixture was stirred for a few min to get a homogenized mixture and then was kept at 70 °C for 90 min to proceed the reaction. After the stated time, methanol was added to cool down the sample and to form precipitates. Precipitated material was washed repeatedly with methanol to remove unreacted TGA. Thereafter, it was cooled at −80 °C and, finally lyophilized at −47 °C and at 0.013 mbar pressure to get dried product. Dried material was kept in a well closed plastic vial at room temperature for further use.

### Evaluation of TARX

#### Fourier transform infrared spectroscopy (FTIR)

FTIR spectra were recorded on Agilent Carry 360 FTIR (United States) to observe any change in the chemical structure as well as to confirm the thiol modification of ARX. Samples of ARX and TARX were scanned in the range of 500 cm^−1^ to 4000 cm^−1^ using KBr technique.

#### Differential scanning calorimetry (DSC)

ARX and TARX were subjected to DSC by using differential scanning calorimeter (DSC-60A Thermal analyzer, Shimadzu Japan). 7 mg sample of each, ARX and TARX was weighed accurately and sealed in the aluminum pan. After that the samples were heated at the rate of 10 °C/min from 35 °C to 300 °C. Nitrogen containing atmosphere was provided while flow rate was adjusted to 25 ml/min [19].

#### X-ray diffractometry (XRD)

To study the crystalline or amorphous structure of the powders and to detect any change in the nature of the polymer in film formulation, samples of both ARX and TARX were analyzed by powder X-ray diffractometry by using X-Rays Diffractometer (JDX-3532 JEOL Japan). Tube voltage was adjusted at 45 kV, tube current 40 mA and scanning angle 2θ was 5–50° [19].

#### Surface morphology

Surface morphology was studied by using Scanning Electron Microscopy (SEM) (JOEL Analytical Scanning Electron Microscope (JSM-6490A, Tokyo Japan).

SEM of ARX and TARX was done for their comparative analysis and to observe any change in the apparent morphology upon conversion of ARX into TARX. Small sample of both ARX and TARX was placed over glass slide and then mounted over the stage of microscope thereafter; SEM was performed to observe the surface morphology at micro level of ARX and TARX by using lens of 2500X resolution power.

#### Determination of thiol contents

Number of thiol contents per gram of the TARX were determined by the quantification of thiol substituents using the Ellman’s reagent method [20, 21]. 0.5%, w/v dispersions of ARX (control) and TARX (sample) were prepared and diluted with 0.5 M phosphate buffer of pH 8.0 to a concentration of 0.15% w/v. 5 ml sample volume of the polymer solution was reacted with equal volume of 0.3% w/v solution of the Ellman’s reagent for 2 h at room temperature. After that, reaction mixture was analyzed by using double beam UV-visible spectrophotometer (PG instruments T80, UK) and the absorbance was recorded at 450 nm. The numbers of thiol constituents per gram of TARX were determined by using a calibration curve prepared by reacting standard solutions of TGA with Ellman’s reagent as earlier detailed.

### Preparation of mucoadhesive oral films

The mucoadhesive ability of TARX was assessed by fabricating TZN HCl-containing controlled release mucoadhesive oral films. CCRD was applied in the design and optimization of films by using Design Expert software (Version 7.1.6, Stat- Ease Inc, Minneapolis, MN) and selecting two variable TARX (X_1_) as a film former and glycerol (X_2_) as a plasticizer (Table 1).

**Table 1:** Independent variables and their different levels used in the formulation of Tizanidine HCl films

Name	Variables	Xmin	Xmax	-β	−1	0	1	+β
TARX (mg)	X_1_	200	300	179.2893	200	250	300	320.7112
Glycerol (%)	X_2_	15	25	12.9289	15	20	25	27.0711

Initially, 13 formulations (Table 2) were designed and 9 out of these formulations were processed. Finally 3 formulations, F3, F5 and F8 were optimized and subjected to various characterizations.

**Table 2:** Composition of Tizanidine HCl films according to CCRD

Formulations	TARX	Glycerol
	Mg	mg
F1	300	45
F2	200	30
F3	250	37.5
F4	250	37.5
F5	300	45
F6	200	30
F7	179.3	26.9
F8	320.7	48.1
F9	250	37.5

Constant concentrations of HPMC K15M (2 ml of 2% solution) polysuccralose (1 ml of 2% solutions) were used in all the formulations

Controlled release mucoadhesive buucal films of TZN HCl were prepared according to the composition of the formulations, described in Table 2. For ease in preparation, w/v aqueous solutions of all the ingredients were prepared. Solution of TARX was 3%, glycerol 6%, HPMC K15M 2%, TZN HCl 3% and, of polysucralose was 2%. First of all, required volume of TARX was takenin a beaker and stirred by using hot plate magnetic stirrer (JISICO J-HSD180 Korea) after that glycerol, HPMC K15M, TZN HCl and sweetening agent were added one by one in the beaker and stirred for half an hour. After confirming complete mixing of all gradients, the mixture was poured in a glass petri dish having a surface area of approximately 24 cm^2^ and an inverted funnel was placed over it. Petri dish was placed in hot air oven at 40 °C for 24 h to get dried films. Dried films were peeled of by using a sharp edge knife, wrapped in aluminum foil and kept in a desiccator at room temperature for further use.

#### Numerical optimization and data analysis

Three responses, mucoadhesion strength, in-vitro percentage drug release (%DR) and ex-vivo drug permeation (DP) were studied by using the Design Expert Software (Version 7.1.6, Stat- Ease Inc, Minneapolis, MN) and analyzed by analysis of variances (ANOVA). Polynomial equation was constructed and interaction and quadratic factors were studied for all the responses, using multiple linear regression analysis (MLRA) approach. The mathematical form of the MLRA model is given as [22].

1 $$ \mathrm{Y}={\upbeta}_0+{\upbeta}_1{\mathrm{X}}_1+{\upbeta}_2{\mathrm{X}}_2+{\upbeta}_{12}{\mathrm{X}}_1{\mathrm{X}}_2{{\hbox{-} \upbeta}_1}^2{{\mathrm{X}}_1}^2{{\hbox{-} \upbeta}_2}^2{{\mathrm{X}}_2}^2 $$

Where, βo is the intercept that describes the mathematical mean of all numerical outcomes of all the trials, β_1_ and β_2_ were the coefficients and they were calculated from the observed experimental values of Y, and X_1_ and X_2_ were coded levels of the independent variable(s). The terms symbolizing the interaction and quadratic terms of the study were represented as X_1_X_2_ and Xi^2^ (i = 1 to 2) respectively. 3-D response surface graphs and 2-D contour plots were drawn by using the output files created to investigate the responses [23, 24].

### Characterization of mucoadhesive oral films

#### Thickness of the films

Thickness can help to predict the uniformity of the drug contents [25]. Piece of films with 2 × 2.5 cm^2^ dimensions were cut and thickness at three different positions was measured by using digital micrometer (H-2780, Mitutoyo Digital Micrometer USA) with least count 0.01 mm. The values were recorded as mean and standard deviations [26].

#### Folding fortitude of the films

Folding fortitude is the measure of mechanical strength and resistance of a film to break upon repeated folding. It was calculated by folding the strip, again and again at the same point until it breaks at that point and number of folds were counted and recorded [27]. Values were calculated as mean and standard deviation.

#### Weight variation test of the films

Uniformity of the weight is an important parameter as it is greatly linked with the uniformity of the drug contents in the film formulation [28]. 4 pieces of films having 2 × 2.5 cm^2^ were cut and weighed accurately on digital electronic weighing balance in triplicate manner. Results were recorded as mean and standard deviation [29].

#### Surface pH of the films

pH is very important as compatibility of formulation pH with the pH of buccal cavity is essential for patient compliance. Surface pH of the films was measured by using 25CW microprocessor bench top pH/mV meter (BANTE instruments, China). The surface of the film was moistened by pouring few drops of deionized water and electrode of pH meter was brought in contact with moist film surface, until the pH value was stabilized. Process was repeated for three time and the readings were recorded as mean and standard deviation [30].

#### Percent moisture contents of the films (%MC)

Four pieces with the dimensions 2 × 2.5 cm^2^ from each formulation were cut and weighed individually on digital weighing balance. After that, they were placed in hot air oven (DHIAN Lab Tech Korea) at 50 °C until the constant weight was achieved. After drying their final weight was noted and then percent moisture contents were calculated as;

$$ \%\ \mathrm{Moisture}\ \mathrm{Contents}=\frac{\mathrm{Wo}-\mathrm{Wd}}{\mathrm{Wo}} \times 100 $$

Where; Wo is initial weight and Wd is the weight after drying.

#### Percent drug contents of the films

Percent drug contents were calculated by dissolving a piece of 2 × 2.5 cm^2^ in 100 ml phosphate buffer of pH 6.8 under continuous stirring. A suitable aliquot of sample was taken after the complete dissolution of the film and then diluted with the buffer. Absorbance of the sample was observed at 228 nm by using a double beam spectrophotometer (PG instrument T80, UK).

#### Surface morphology studies of films

Visual inspection (VI) and scanning electron microscopy (SEM) were used for the analysis of the surface of the formulated films. VI was done to observe the apparent structure of the film while SEM was used to observe the structure even more deeply at a magnification of 2500X.

#### X-ray diffraction study of powders films

X-RD is a vital parameter to observe the crystalline or amorphous nature of the drug both in powder form as well as in film formulation. XRD was performed by maintaining the experimental conditions as tube voltage 45 kV, tube current 40 mA and scanning angle 2θ was 5–500.

#### Ex-vivo mucoadhesive Strength of the films

The mucoadhesive strength of the TARX-based TZN HCl-containing buccal films was calculated by the reformed physical balance method [17, 31]. This apparatus contained tarred two pan physical balance. Three glass slides were taken; one glued to the bottom of one of the pans, 2^nd^ glass slide was attached at the base beneath the pan and third attached to the other pan, in order to tare the balance. Buccal mucosal membrane (Buccal mucosa of goat was collected from local slaughter house, washed with isotonic phosphate buffer and kept in normal saline till further used) was attached to both slides and a strip of TARX film was sandwiched between the buccal mucosa. 50 g preload force was applied over the pan for 5 min. After that force was removed and balance was elevated by holding the lever. Weight was applied in gradually increasing manner by adding water till the glass slides were isolated from each other. The weight (g) of the water required for separation of slides was noted. Force of detachment (N) was measured and taken as an index of the mucoadhesive strength of films.

#### Construction of calibiration curve of TZN HCl

5 mg of TZN HCl was weighed accurately and transferred to volumetric flask with the capacity of 50 ml. Small volume of phosphate buffer of pH 6.8 was added to dissolve the drug. Finally the volume was made up to 50 ml. The concentration of TZN HCl in this stock solution was 100 μg/ml. From the stock solution, different dilutions of concentration 1 μg/ml-10 μg/ml were prepared and analyzed spectrophotometrically at 228 nm.

#### In-vitro drug release of the films

In-vitro drug release studies were carried out in USP II dissolution apparatus [32, 33]. 500 ml of 6.8 pH phosphate buffer was used as dissolution medium. Paddle speed was adjusted to 50 rpm and the temperature of the apparatus at 37 ± 0.5 °C. Piece of the film with dimensions of 2x2.5 cm^2^ from each formulation was cut and carefully placed on the surface of a glass slide with the help of paper pin. Glass slide holding a strip was carefully dropped into the vessel of dissolution apparatus and dissolution was started. 5 ml of aliquot was drawn after every hour and the same volume of fresh medium was added in order to maintain total dissolution medium 500 ml. Studies were carried out for 8 h and percentage drug release was calculated by analyzing the sample absorbance taken at 228 nm by using double beam spectrophotometer (PG instrument T80).

#### *Ex-vivo* permeation studies of the films

*Ex-vivo* permeation of the drug across goat buccal mucosa was studied with the help of Franz diffusion cell, using phosphate buffer of pH 6.8 as a medium [34]. Buccal mucosa of goat was collected from local slaughter house, washed with isotonic phosphate buffer and kept in normal saline till further use [35]. The volume holding capacity of the Franz cell with the capacity of 13 ml was taken and buccal mucosa was mounted between the receptor and donor compartments of the cell. Strip of the film was placed on the mucosa and then both the compartments of franz cell were clamped together. LP needle was used to draw the sample after every 1 h interval till 8 h of the study. Working conditions of 37 °C ± 0.5 °C and 50 rpm speed of the shaker water bath were maintained throughout the study. Aliquots of 3 ml were drawn at each interval and replaced with same volume of fresh medium to maintain constant volume. Amount of permeated drug was calculated by analyzing the sample absorbance taken at 228 nm using double beam spectrophotometer.

#### Kinetic modeling of the drug release and drug permeation

Kinetic models including zero order, first order, Higuchi model, Hixson crowell cube root and Korsmeyer peppas model were applied to find out the release kinetics and pattern of drug release from the formulated mucoadhesive buccal films of TZN HCl [1].

#### Drug-excipients compatibility

Fourier transform infra-red (FTIR) spectroscopy was used to find out the compatibility of the ingredients and to detect any kind of drug-excipients interaction. FTIR spectrum of TZN HCl, TARX and TARX-films were taken [36].

## Results and discussions

Different parameters were studied to assess the quality of the formulated mucoadhesive films. Average thickness of the films was in the range of 0.147 ± 0.041 to 0.167 ± 0.036, suggesting a link between the amount of the polymer and thickness. Films, having greater amount of the polymer, were found to be more thicker then those having comparatively lesser quantity of the polymer. The same trend was observed while calculating the average weights of the films; but weight variation amongst the films of a same formulation was negligible with standard deviation 0.001 to 0.003. pH of the films was agreeable, having the range between 6.59 ± 0.19 to 6.73 ± 0.41 and was found comparable to the pH of the buccal cavity.

**Fig. 1:**
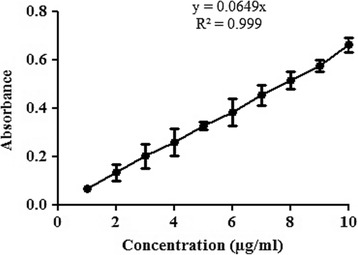
Calibration curve of TZN HCl

Calibiration curve, that was constructed by using different dilutions (1 μg/ml to 10 μg/ml) of drug, showed good linearity with R^2^ 0.999, as shown in the Fig 1. Calibration curve was used to determine the drug content of the film formulation. The drug loading ability of the film was acceptable having the range of % drug contents between 95.42 ± 4.1 to 97.03 ± 2.8%. When the films of selected formulations were subjected to the determination of % moisture contents, it was observed that the films with greater concentration of glycerol, contains comparatively greater amount of the moisture content. Retention of greater moisture content in the films may be due to the hygroscopic nature of the glycerol. Folding endurance is a suitable parameter to judge the mechanical strength of the films. All prepared films had admirable folding endurance, and none showed any cracks below 300. Tensile strength of the films was found variable from 0.31 ± 0.067 (F8) to 0.89 ± 0.017 N (F3). Ex-vivo mucoadhesion was evaluated to judge the ability of the films to remain attached to the mucosal membrane. In the current study, it was observed that mucoadhesion was found to be increasing with increase in polymer concentrations (Table 3).

**Table 3:** Evaluation of TARX based mucoadhesive buccal films containing TZN HCl

Formulations	Average thickness (mm)	Average weight (mg)	Average pH	% Moisture contents	% Drug contents	Folding endurance	Mucoadhesion strength	Tensile strength
F3	0.147 ± 0.041	75.0 ± 0.001	6.59 ± 0.19	1.28 ± 2.1	97.03 ± 2.8	>300	9.23 ± 0.21	0.89 ± 0.017
F5	0.161 ± 0.043	83.5 ± 0.003	6.73 ± 0.41	1.4 ± 1.8	95.42 ± 4.1	>300	10.47 ± 0.19	0.42 ± 0.273
F8	0.167 ± 0.036	96.0 ± 0.002	6.64 ± 0.27	1.21 ± 1.7	95.19 ± 4.9	>300	11.53 ± 0.17	0.31 ± 0.067

**Fig. 2:**
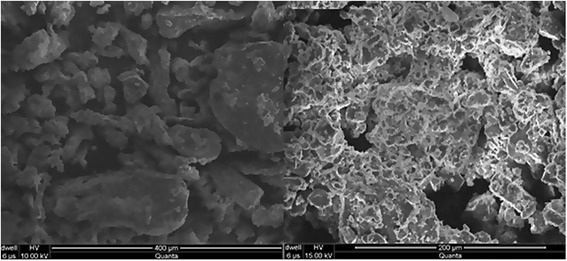
SEM of ARX (left) and TARX (right)

**Fig. 3:**
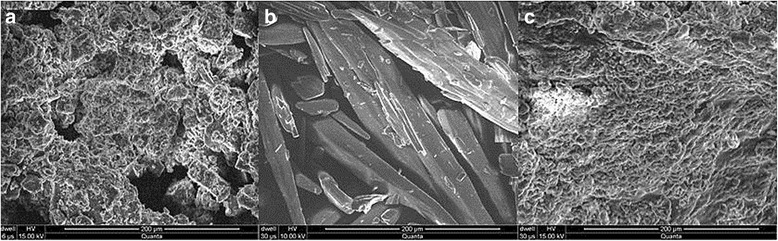
SEM images of TARX (**a**), TZN HCl (**b**) and F5 (**c**)

**Fig. 4:**
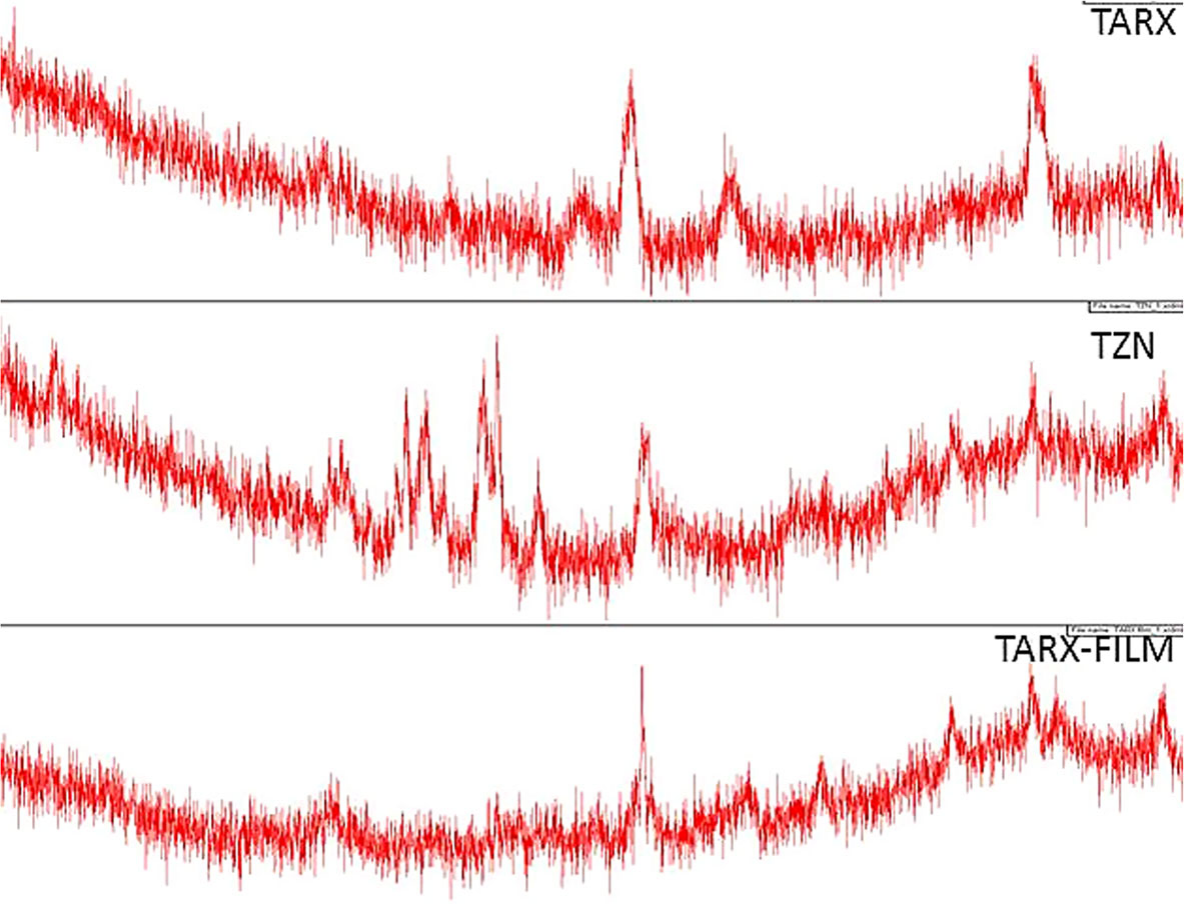
X-RD thermogram of ARX, TARX, TZN HCl and F5

The surface morphology of the ARX, TARX, TZN HCl and formulated mucoadhesive oral film of TARX containing TZN HCl was studied by using SEM. SEM revealed that TARX is a bit crystalline material with irregular shaped particle (Fig. 2) while TZN HCl is a crystalline drug. SEM of the film showed that the drug and polymer were mixed thoroughly, although the surface appeared to be uneven (Fig. 3). When drug, polymer and formulated film were subjected to x-ray diffraction, the crystallinity of the polymer and drug was confirmed by the appearance of different peaks which were diminished in film formulation confirming uniform distribution of the drug and polymer. The diffractogram of TZN HCl exhibited a series of intense peaks at Ɵ 10° to 45° the most indicative were approximately at 10°, 21°, 24°, 25°, 26°, 27°, 28°, 32° and 45° [37, 38]. Diffractgram of TARX showed two distinguishing sharp peaks at 32° and 45° (2$\theta$) while a typical amorphous diffractogram was seen in case of mucoadhesive film (Fig. 4).

### *Ex-vivo* mucoadhesion strength

2 $$ \mathrm{Mucoadhesion}\ \mathrm{strength} = 9.23 + 1.42{\mathrm{X}}_1+0.60{\mathrm{X}}_2\hbox{--} 0.18{\mathrm{X}}_1{\mathrm{X}}_2+0{{.064\mathrm{X}}_1}^2\hbox{--} 0.24{\mathrm{X}}_2 $$

Data on mucoadhesion strength, as evaluated by ANOVA indicated that model terms were significant as the *P* values were 0.0460 (<0.05). R-squared was 0.9411 and Adjusted R-squared was 0.8430. The presence of glycerol decreases the crosslinking density of the polymer network leading to rise in the estimated mucoadhesiveness. Additionally, it was described that glycerol has three folds the hygroscopicity of polyethylene glycol (PEG 600). Therefore, glycerol increases the fluidity of the dosage form, as a result of the hydration, adhesion might be promoted by affinity to dehydrate an intermediate mucin layer [39]. In recent times, it has been revealed that polymers with thiol groups offer much greater adhesive properties than polymers, usually considered to be mucoadhesive. The improvement of mucoadhesion can be clarified by the formation of covalent bonds between the polymer and the mucus layer, which are stronger than non-covalent bonds [40].

**Fig. 5:**
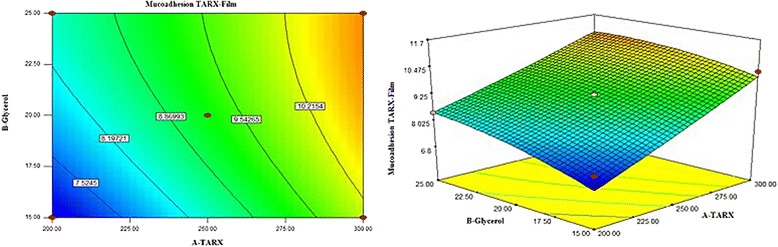
Contour and 3D graphs showing effect of TARX and glycerol on mucoadhesion strength of the films

RSM 3-D and 2-D contour plots are drawn in Fig. 5, expressing the relationship of X_1_ and X_2_ and their effect on mucoadhesion strength of the mucoadhesive buccal films. Figure 5 explained that both variables X_1_ and X_2_ are found to be effective in increasing adhesiveness of the films. Polynomial equation 2 exposed constructive effect of the factors. The mean of the dependent variable is a positive value (9.23), which advocated that response is positive and both factors have a significant influence on adherence of film on mucosal membrane. In equation, both factors (X_1_ and X_2_) have positive values (+1.42 and +0.60) which is the indication that they are quite capable of improving the bonding strength between the film and mucous membrane. Quadratic term X_1_^2^ showed a positive response indicating a direct relation between concentration of the TARX and extent of mucoadhesion strength. Hence, it is proved that mucoadhesion can be enhanced by increasing the concentration of mucoadhesive polymer (TARX) to a suitable extent.

### *In-vitro* dissolution studies

3 $$ \%\ \mathrm{Drug}\ \mathrm{Release} = 97.03\hbox{--} 5.77{\mathrm{X}}_1+6.37{\mathrm{X}}_2+3.95{\mathrm{X}}_1{\mathrm{X}}_2\hbox{--} 2{{.97\mathrm{X}}_1}^2\hbox{--} 3{{.54\mathrm{X}}_2}^2 $$

Data of % drug release was evaluated by ANOVA and it was found that model terms are not significant as the *P* values are 0.1053 (>0.05). R-squared was 0.8946 and Adjusted R-squared was 0.7190..

**Fig. 6:**
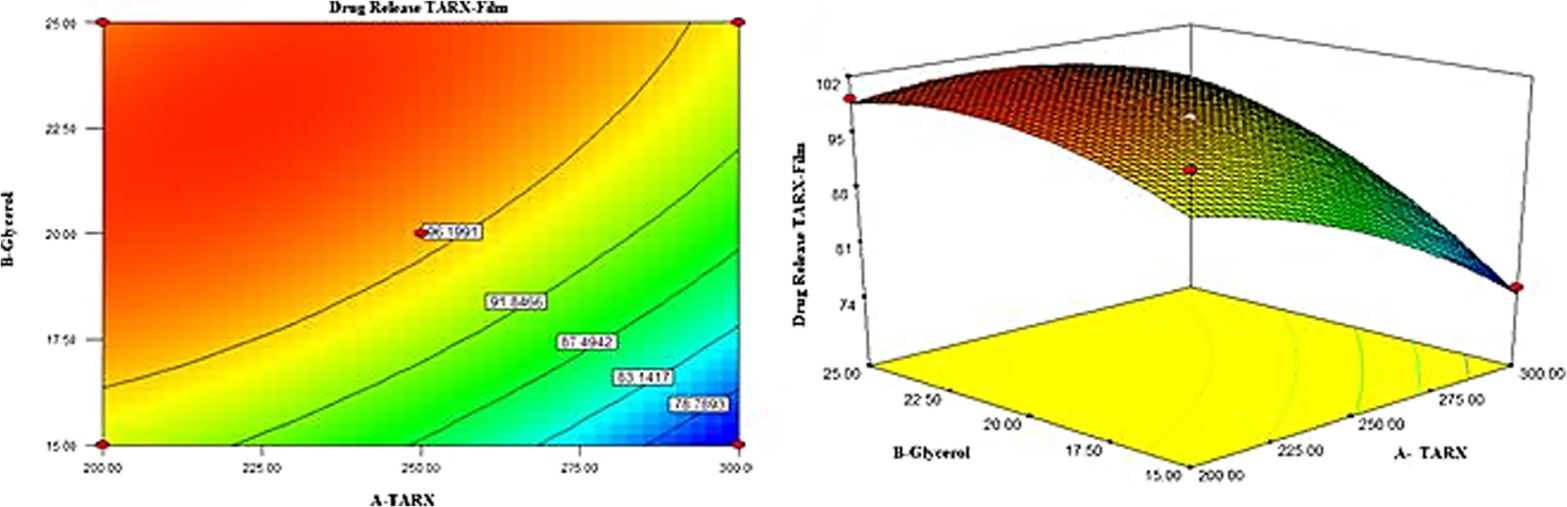
Contour and 3D graphs showing effect of TARX and glycerol on the release of the TZN HCl from films

The 3-D graph was shown in the Fig. 6, stating the effect of TARX and glycerol. Polynomial equation 3 showed that increase in X_1_ can reduce the extent of drug release whereas the increase in X_2_ will heighten the drug release from the mucoadhesive buccal films. Both interacting factor X_1_X_2_ have positive values that mean simultaneous variation in both X_1_ and X_2_ extend the dissolution of the films. Quadratic terms were found to be decreasing the amount of drug released from the films.

**Fig. 7:**
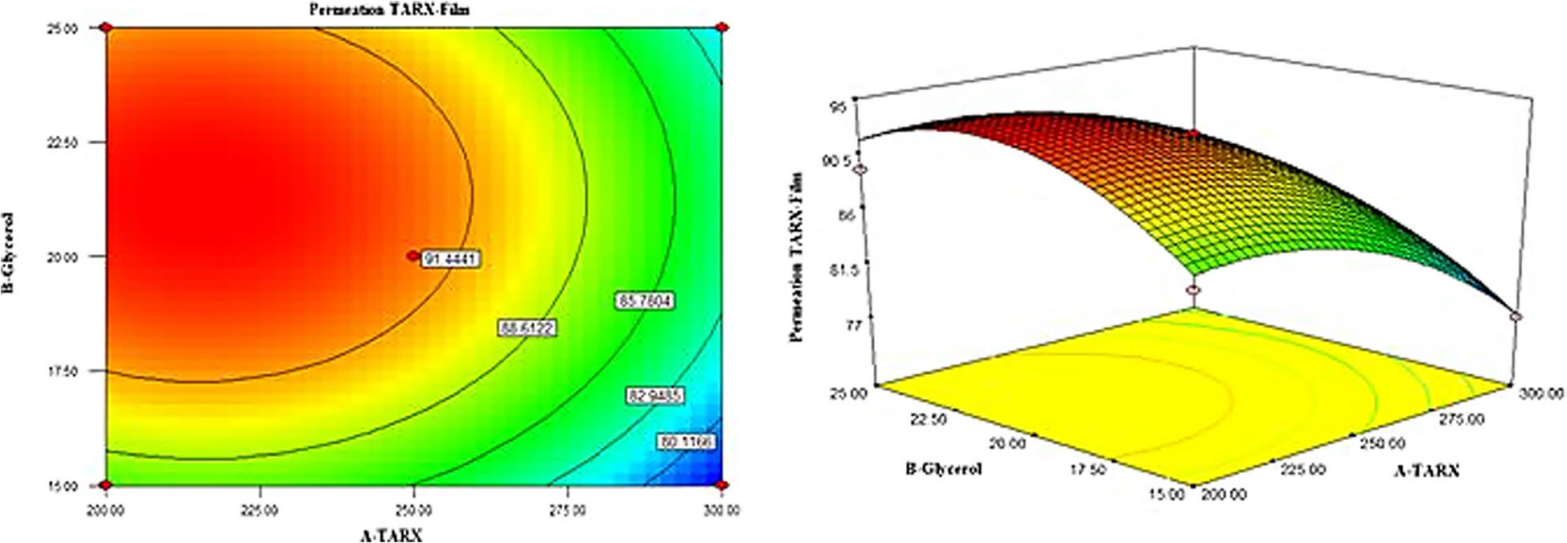
Contour and 3D graphs showing effect of TARX and glycerol on drug permeation across the goat buccal mucosa

The presence of glycerol as plasticizer in a film formulation, supports the plasticity and elasticity of the film, moreover, they are effective in enhancement of drug dissolution as well as permeation. Because of the hygroscopicity of glycerol, an increase in the water uptake by mucoadhesive film from the release medium increases the dissolving medium available for the drug. Moreover, glycerol may cause the reduction in the viscosity of the polymeric gel; hence, as the TARX is a gel forming and swellable polymer, it was anticipated that, it can enhance the rate of drug release from the films. This may be the clarification of comparatively increased drug release from the formulations containing greater amount of glycerol (Fig. 7).

**Fig. 8:**
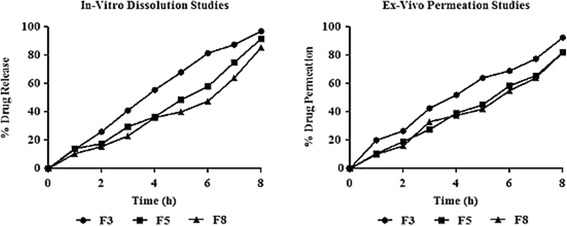
Graphs showing a pattern of drug release and drug permeation from the films

Earlier, it was pointed out that the thickness of the gel layer controls the drug release [39]. TARX, being a swellable and gel forming polymer retards the drug release from film formulation by forming a gel layer around the drug particle. As the actual passageway for drug penetration across the gel layer is the liquid medium captured in the gel pores, so, the factors that affect diffusivity in pure liquid phases can control penetration within gels. Therefore, the thickness of the gel layer could be the important factor, which influences the release of drug from films. Thickness of the gel layer is directly linked with the polymer concentration, hence, the formulations that had greater amount of polymer released lesser amount of the drug, indicating that increase in polymer concentration will enhance the drug retarding ability of the controlled release formulation [13, 41]. Similar kind of situation can be observed from Fig. 8 and polynomial equation (3), describing the effect of TARX and glycerol in drug release from the mucoadhesive films.

Selected formulations, F3, F5 and F8 have released 97.03, 91.21 and 85.13% of the drug respectively. Results of in-vitro drug release studies described that selected formulations are quite suitable for delivering suitable amount of drug in controlled manner.

### *Ex-vivo* drug permeation

4 $$ \mathrm{Drug}\ \mathrm{permeation}\ \left(\mathrm{DP}\right) = 92.22\hbox{--} 4.97{\mathrm{X}}_1+2.24{\mathrm{X}}_2\hbox{--} 0.068{\mathrm{X}}_1{\mathrm{X}}_2\hbox{--} 3{{.52\mathrm{X}}_1}^2\hbox{--} 4.28{\mathrm{X}}_2 $$

Drug permeation across buccal mucosal membrane can be a limiting factor for numerous drugs given through the buccal route. Drug permeation enhancers are capable of reducing permeation hurdle of the mucosa membrane. A reversible method of reducing the barrier potential of mucosal tissues may be applied to deliver a large number of drugs through this route. The permeation enhancing agent can change the potential of this obstacle safely, causing an increase in drug permeation. Different mechanisms can be adopted by the permeation enhancer to improve the transport of drug across the mucosal membrane and these mechanisms may include; increasing cell membrane fluidity, extracting intercellular lipids, interacting with epithelial protein domains, altering mucus structure and rheology [42]. Glycerol can enhance the infiltration of drug across the skin as well as mucosal membranes [43]. Glycerol has the ability to penetrate into the membrane and maintain the penetrability characteristics related with a hydrated membrane, even in circumstances of low water activities, which else would dehydrate the membrane and can restrict the penetrability [44].

In order to assess the influence of TARX and glycerol, permeated data as evaluated by ANOVA revealed that model terms are significant as the *P* values are 0.0459 (<0.05). R-squared was 0.9412 and Adjusted R-squared was 0.8431.

RSM 3-D and 2-D contour plots are drawn in Fig. 7, expressing the relationship of X_1_ and X_2_ and their effect on DP. It is clear from the graphs that the increase in X_1_ will be the cause of decrease in drug permeation, however, X_2_ has the opposite effect and increase in its concentration will enhance the drug permeation through the buccal mucosa. Polynomial equation 2 revealed that the mean of the dependent variable is a positive value, which suggested that the response is positive and both factors have significant influence on the DP. Quadratic term X_1_^2^ showed a negative response as well as interacting factors X_1_X_2_ were found to be decreasing the permeation of the drug.

92.22, 80.06 and 78.90% of the drug was found to be permeated across the goat buccal mucosa from the selected formulations F3, F5 and F8 respectively. Here, it can be observed that, as the amount of the polymer was increased, the % of the permeated drug was decreased. However, from the results, conclusion can be drawn that a controlled release formulation with suitable drug permeating ability can be developed by adjusting appropriate concentrations of the film former and plasticizer. In the current study, 1:6.66 of plasticizer to polymer was found suitable for formulation of controlled release mucoadhesive oral film.

### Kinetic modeling of drug release and drug permeation

Dissolution data was analyzed for mechanism and behavior of drug release by different kinetic models. All three formulations (F3, F5 and F8) showed a variable behavior and mechanism of drug release as it was observed that zero order, Korsmeyer- peppas model and Hixon- crowel cube root were the best fit models for F3, F5 and F8 respectively. However, there were greater values of R^2^ for zero order kinetics as compared to the 1^st^ order kinetics, suggesting that release of the drug was controlled and independent of initial concentration of the drug in the films.

Kinetics of the permeated drug were also observed by the kinetic models and it was seen that drug permeation of F3 and F8 was following zero order kinetics while F5 was following the Korsmeyer- peppas model. Values of ‘n’ obtained from korsmeyer-peppas model suggested that the release of the drug from the mucoadhesive films was following super case II transport mechanism as the *n >* 1. The only exception was observed in case of F3, when data was analyzed, it showed non-fickian diffusion pattern as the value of n was less than 1 [45, 46].

Drug permeation was also controlled and independent of initial concentration of the drug in the film as R^2^ for zero order kinetics was greater than 1^st^ order kinetics. R^2^ of Hixson crowell cube root was also high (0.9447 to 0.9731), ensuring a uniform exhaustion of the film surface during permeation studies (Table 4).

**Table 4:** Kinetics modeling of released and permeated drug from mucoadhesive oral films

Kinetic models		In-vitro dissolution studies			Ex-vivo permeation studies		
		F3	F5	F8	F3	F5	F8
Zero order	R^2^	0.9858	0.9692	0.9407	0.9525	0.9929	0.9804
	k^o^	12.853	10.400	9.129	11.844	9.600	9.337
First order	R^2^	0.9150	0.8535	0.8407	0.9481	0.9247	0.9184
	K^1^	0.227	0.154	0.127	0.202	0.139	0.134
Hixson- crowell cube root	R^2^	0.9603	0.8943	0.8747	0.9731	0.9538	0.9447
	kHC	0.064	0.045	0.038	0.057	0.041	0.040
Higuchi model	R^2^	0.8468	0.7389	0.7002	0.8906	0.7939	0.7874
	kH	30.193	24.071	21.037	28.062	22.360	21.761
Korsmeyer- peppas model	R^2^	0.9924	0.9830	0.9671	0.9880	0.9940	0.9811
	kKP	15.391	7.307	5.339	17.233	8.815	8.711
	n	0.900	1.194	1.295	0.791	1.047	1.038
Best fit model		Zero order	Korsmeyer- peppas model	Hixson- crowell cube root	Zero order	Korsmeyer- peppas model	Zero order

### Compatibility studies

The covalent attachment of TGA acid to the hydroxyl group of arabinoxylan was achieved by the ester bond formation between hydroxyl group of arabinoxylan and carboxyl group of TGA.

**Fig. 9:**
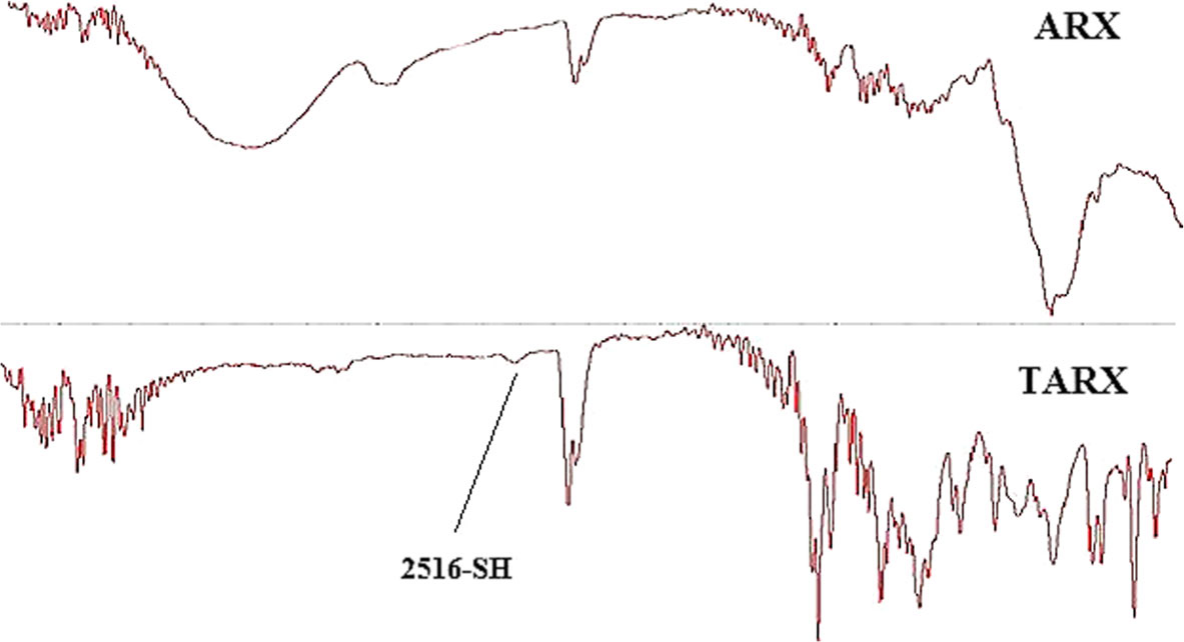
FTIR of ARX and TARX

Figure 9 displays the FT-IR spectrum of Arabinoxylan and thiolated arabinoxylan in the frequency range from 4000 to 500 cm^−1^. In case of arabinoxylan a broad absorption band can be seen at 3321 cm^−1^ which can be accredited to –OH stretching of alcohols. A peak appeared at 2931 cm^−1^ due to –CH stretching of alkanes. The peak at 1030 cm^−1^ was recognized as C–O–C stretch of ether. The other peaks observed at 895, 717, and 609 cm^−1^ were due to polymer backbone bending.

The IR spectrum of thiolated arabinoxylan reveals all the important absorption bands, sufficient to prove the ester linkage between arabinoxylan and TGA.

**Fig. 10:**
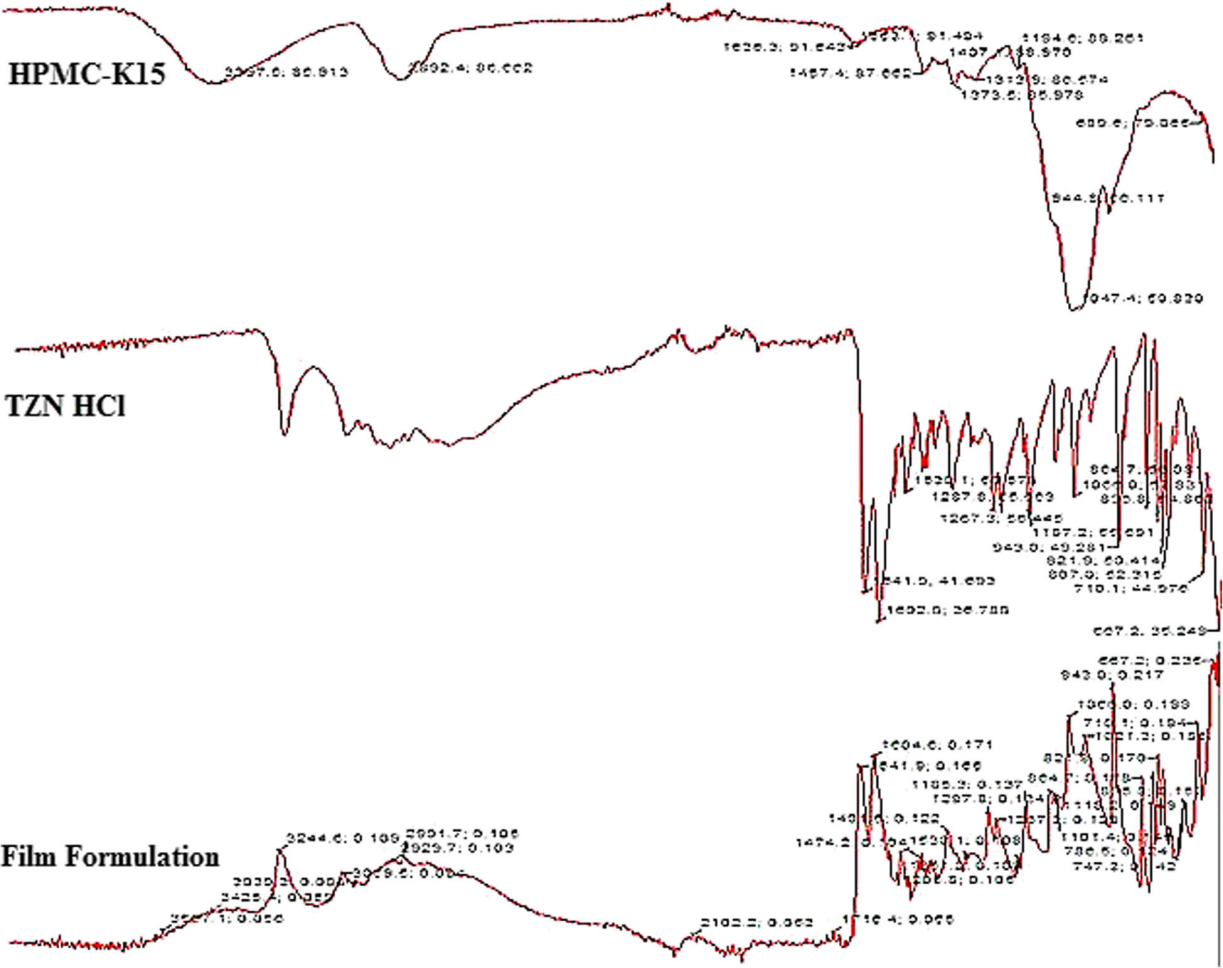
FTIR spectrum of HPMCK15M, TZN HCl and F5

The key functional groups, including the C = O of ester and –SH stretch appeared at 1710 cm^−1^ and 2516 cm^−1^, respectively. The C–O–C stretch appeared at 1031 cm^−1^. The stretch appeared at 1031 cm^−1^ while the peaks at 892, 771, and 643 cm^−1^ could be attributed to polymer backbone bending. The other related stretching frequencies were also observed in the appropriate region of infra-red spectra. All the FTIR bands were found consistent with those observed previously in the literature. In the FTIR spectrum of pure drug, characteristic peaks were observed in the desired range such as 1086 cm^−1^ C-N stretching in amines, 1530 cm^−1^ C = C stretching, 1602 cm^−1^ C = N stretching (in ring), 2770 cm^−1^ C-H stretching in CH_3_, CH_2_ and 3250 cm^−1^ N-H stretching in primary amines [47, 48]. FTIR spectrum of HPMC K15M showed characteristics peak at 1047 cm^−1^ for CO stretch and bending vibration of CH_3_ and CH_2_ were observed at 1373 cm^−1^ and 1457 cm^−1^ respectively..Bands near 3425 cm^−1^ (O-H stretching vibration) and peak at 2892 cm^−1^ was due to the C-H stretching vibration. FTIR spectrum of formulated mucoadhesive film showed all characteristic peaks with negligible variation or shift in the characteristic peaks of the ingredients, suggesting that the selected combination of the ingredients is suitable and has no chemical interaction between each other (Fig. 10).

## Conclusion

Results and above discussion suggest a successful completion of the project as the goal of thiolation of ARX and development of controlled release mucoadhesive oral film of TZN HCl was achieved quite successfully. Films were fabricated with reasonable mucoadhesion strength and good control over release and permeation of the drug. It was also concluded that both film former and plasticizer have pronounced impact on the quality of the dosage form. Selection of suitable and compatible ingredients is very important and the more important thing is the adjustment of suitable quanities of the excipients. Glycerol has the ability to enhance the elasticity, dissolution profile as well as the permeability of the drug. On the other hand TARX posess the ability of sustaining the drug in the reservoir. In this study, statistical approach was used effectively in optimizing the formulation and finilazing 1:6.66 ratio of plasticizer to the polymer for controlled release mucoadhesive oral films of TZN HCl. Finally, the current project was accomplished successfully and in nutshell, the method adopted for thiolation of arabinoxylan and selection of other excipients to support TARX for the development of controlled release mucoadhesive oral film formulation is declared appropriate.

## Abbreviations

ANOVA: Analysis of variances
ARX: Arabinoxylan
CCRD: Central composite rotatable design
FTIR: Fourier transformed infra-red spectroscopy
GIT: Gastro intestinal tract
NSAIDs: Non-steroidal anti-inflammatory drugs
SEM: Scanning electron microscopy
TARX: Thiolated arabinoxylan
TGA: Thioglycolic acid
TZN HCl: Tizanidine hydrochloride
X_1_: Variable factor 1 that is thiolated arabinoxylan
X_2_: Variable factors 2 that is glycerol
XRD: X-ray diffraction
